# Well-Known and Novel Behavioural Risk Factors for Heart Failure

**DOI:** 10.3390/jcdd13050211

**Published:** 2026-05-14

**Authors:** Natalia Kusyn, Natalia Zdebik, Wojciech Hajdusianek, Rafał Poręba, Paweł Gać

**Affiliations:** 1 Department of Environmental Health, Occupational Medicine and Epidemiology, Wroclaw Medical University, Mikulicza-Radeckiego 7, 50-345 Wrocław, Poland; 2 Lower Silesian Mental Health Centre, J. C. Korzeniowskiego 18, 50-226 Wrocław, Poland; 3 Department of Biological Principles of Physical Activity, Wroclaw University of Health and Sport Sciences, I. J. Paderewskiego 35, 51-612 Wrocław, Poland

**Keywords:** heart failure (HF), heart failure risk factors, behavioural HF risk factors

## Abstract

Heart failure (HF) is a clinical syndrome characterized by structural or functional cardiac abnormalities that impair ventricular filling or ejection, leading to inadequate systemic perfusion and elevated intracardiac pressures. Current epidemiological estimations declare approximately 26 million patients affected worldwide are living with HF. While ischemic heart disease remains the primary etiology, there is a wide range of behavioural factors that significantly influence disease onset and progression. This review focuses on the evidence for established risk factors, including smoking, excessive alcohol consumption, obesity, physical inactivity, poor diet, sleep disorders, and psychological stress. Furthermore, we discuss other novel determinants such as electronic nicotine delivery systems (ENDS), cannabis, high-dose caffeine, and psychostimulants. The basic mechanistic pathways, including endothelial dysfunction, oxidative stress, neurohormonal activation, and direct myocardial toxicity, are also pointed out and reviewed in this paper. The aim of this study is to integrate epidemiological data with pathophysiological insights to identify priority targets for primary prevention and highlight areas for future research.

## 1. Introduction

**Figure 1:**
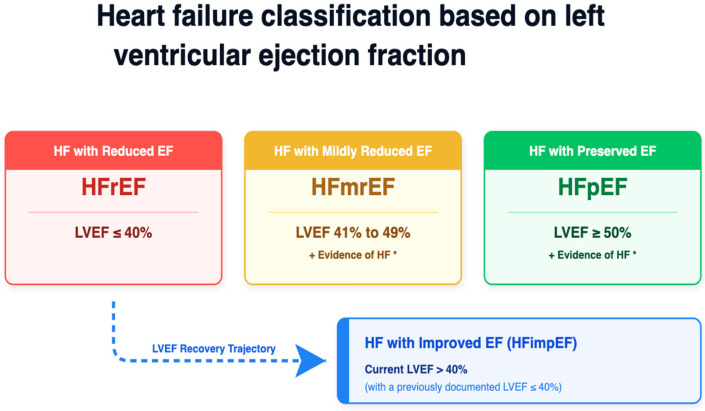
Classification of heart failure based on left ventricular ejection fraction (LVEF). Patients are categorized into three primary phenotypes: HFrEF, HFmrEF, and HFpEF. A fourth, dynamic trajectory represents patients with improved ejection fraction (HFimpEF), indicating myocardial recovery. * Evidence of HF: Requires the presence of spontaneous or provocable elevated cardiac biomarkers or elevated filling pressures. Abbreviations: EF, Ejection Fraction; HF, Heart Failure; HFrEF, Heart Failure with reduced Ejection Fraction; HFmrEF, Heart Failure with mildly reduced Ejection Fraction; HFpEF, Heart Failure with preserved Ejection Fraction; HFimpEF, Heart Failure with improved Ejection Fraction; LVEF, Left Ventricular Ejection Fraction.

Heart failure (HF) is a complex clinical syndrome characterized by structural or functional cardiac abnormalities that impair ventricular filling or blood ejection, which leads to inadequate systemic perfusion and elevates intracardiac pressures [1,2,3,4]. Current epidemiological estimations declare approximately 26 million patients affected worldwide [1,3,5]. Coronary artery disease causing ischemic heart disease remains the leading cause of HF in most populations [1]. Furthermore, several modifiable risk factors such as hypertension, diabetes, obesity, smoking, and sedentary lifestyle play a key role in the development and progression of this disease [1]. The left ventricular ejection fraction is used to classify heart failure as shown on Figure 1. This review summarizes the epidemiology of HF and discusses the pathophysiological links between its primary causes and risk factors, highlighting the path of future research required on this topic.

## 2. Materials and Methods

A comprehensive literature search was performed on PubMed and Scopus. The search strategy included various key terms such as heart failure, HF risk factors, and lifestyle determinants, in combination with key terms such as tobacco smoking, electronic nicotine delivery systems, e-cigarettes, alcohol consumption, illicit drugs, cocaine, cannabis, caffeine, sleep disorders, psychological stress, obesity, dietary patterns, physical activity and vitamin deficiencies. We chose and reviewed studies written in English that involved adult populations, with a particular focus on randomized controlled trials, meta-analyses, and large observational studies. Reference management and citation were conducted using Zotero (version 7.0, Corporation for Digital Scholarship, Vienna, VA, USA). The full texts were screened and selected, and the study characteristics and information were extracted from the selected literature to summarize current evidence on behavioural determinants of heart failure. Whenever possible, quantitative effect estimates (relative risks, hazard ratios, odds ratios, and population-attributable risk) and dose–response data were extracted to allow comparison across exposures. Canva (https://www.canva.com, Canva Pty Ltd., Sydney, Australia) and Claude (version 3.5 Sonnet, Anthropic PBC, San Francisco, CA, USA) were used to prepare the figures shown in this paper.

### Assessment of Evidence Quality

To help the reader weigh each association, we used a simplified evidence-grading scheme based on the Level of Evidence (LoE) system. We used three categories: Level A which includes multiple large randomized controlled trials (RCTs), Mendelian-randomization analyses, or high-quality meta-analyses with consistent results; Level B with limited randomized data or large prospective cohort studies; and Level C which contains mainly smaller observational studies, case series, mechanistic studies, or expert consensus. The assigned grade is reported in Table 1 next to the predominant study design and quantitative effect estimates for each behavioural risk factor.

## 3. Pathophysiology of Heart Failure

It is believed that coronary artery disease causing ischemic heart disease is the primary etiology of heart failure. A characteristic of heart failure is that it is a progressive condition, which often starts with an acute damage of the cardiac structure or a change caused by ischemia, valvular disease, or myocarditis. First, there is a wide range of compensatory mechanisms that help to maintain cardiac output, therefore the circulation flows, despite the damages in the heart. These mechanisms are the activation of the sympathetic nervous system (SNS) and as a consequence the renin–angiotensin–aldosterone system (RAAS). However, the long-term activation of the SNS makes beta-receptors less responsive and reduces adrenaline stores, which leads to changes in myocyte regeneration ability and causes myocardial hypertrophy [29,30]. Furthermore, angiotensin II released by the RAAS increases cellular hypertrophy and fibrosis, which drives myocardial remodelling [29]. When cardiac output decreases, the neuroendocrine system responds by releasing epinephrine, endothelin-1 (ET-1), and vasopressin, which increases afterload through vasoconstriction. Elevated levels of cyclic adenosine monophosphate (cAMP) cause an elevation of cytosolic calcium levels in the myocytes, which not only increases contractility but also prevents the heart from relaxing properly. This combination of high afterload, high contractility, and poor relaxation raises the heart’s oxygen demand [29,30]. Eventually, the heart cannot meet its own metabolic needs, which leads to cell apoptosis. The loss of cardiac cells reduces the ejection fraction (EF), resulting in the left ventricle (LV) being unable to empty completely. The resulting increase in LV volume and pressure leads to pulmonary congestion, and low perfusion in the kidneys triggers the release of antidiuretic hormone, which increases water retention. High central venous pressure also reduces renal blood flow, which further decreases the glomerular filtration rate (GFR) [29].

**Figure 2:**
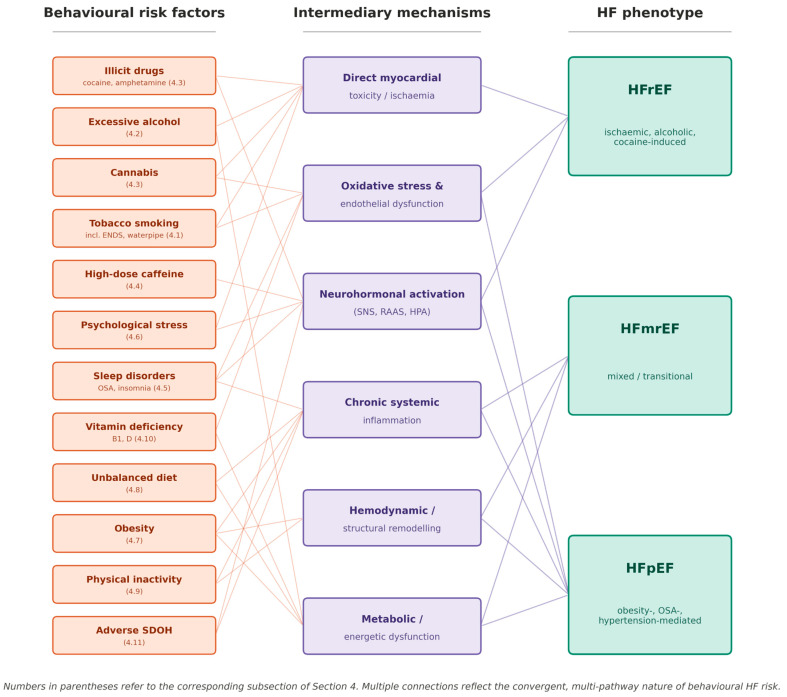
Behavioural risk factors, intermediary mechanisms, and resulting heart failure phenotype. Behavioural risk factors (**left column**) act through intermediary pathophysiological pathways (**centre column**): direct myocardial toxicity and ischemia, oxidative stress and endothelial dysfunction, neurohormonal activation involving the SNS, RAAS and HPA axis, chronic systemic inflammation, hemodynamic and structural remodelling, and metabolic and energetic dysfunction. These mechanisms result in three heart failure phenotypes (**right column**): HFrEF, HFmrEF, or HFpEF. Numbers in the left column refer to the corresponding subsection of Section 4. Multiple connections from each risk factor show the multi-pathway nature of behavioural HF risk factors. Abbreviations: HFmrEF, heart failure with mildly reduced ejection fraction; HFpEF, heart failure with preserved ejection fraction; HFrEF, heart failure with reduced ejection fraction; HPA, hypothalamic–pituitary–adrenal axis; OSA, obstructive sleep apnea; RAAS, renin–angiotensin–aldosterone system; SDOH, social determinants of health; SNS, sympathetic nervous system.

Decompensated heart failure means strong peripheral vasoconstriction and high preload. Although the heart cells produce natriuretic peptides like BNP (brain natriuretic peptide) and ANP (atrial natriuretic peptide), they eventually become ineffective at decreasing sodium and water retention [29]. Figure 2 shows the behavioural risk factors discussed in Section 4 with the intermediary mechanisms and the predominant heart failure phenotype.

## 4. Risk Factors for Heart Failure

It is believed that coronary artery disease causing ischemic heart disease is the primary etiology of heart failure [1]. Yet, there is a wide range of behavioural factors that significantly influence the HF onset and progression. While established HF risk factors include smoking, excessive alcohol consumption, obesity, physical inactivity, unbalanced diet, sleep disorders, and stress exposure, other determinants such as electronic nicotine delivery systems, vitamin deficiencies, cannabis, psychostimulants, and high-dose caffeine require further attention. In Table 1 we summarized the established and emerging risk factors, along with their pathophysiological mechanisms and clinical impacts. In Table 2 we collected data for quantitative effect estimates for behavioural HF risk factors.

### 4.1. Tobacco Smoking

Tobacco use is an established risk factor for various diseases including cardiovascular [1]. This has been proven in different studies for example in the Framingham Heart Study [1]. Moreover, about 16% of all deaths among men (in Europe 25%) and 7% among women are associated with smoking worldwide and these deaths could have been avoided. According to the World Health Organization (WHO), global tobacco consumption decreased significantly over the past two decades. This was due to a decrease in the number of smoking women [31]. However, a new trend for using electronic cigarettes and water pipes (Shisha) simultaneously emerged and became particularly popular among the youth. This trend may be encouraged by the misconception that these products are significantly less harmful or healthier than regular smoking [32,33]. Nicotine—an alkaloid with psychoactive effects—is a reason a person reaches for a cigarette.

It should be emphasized that there is no such thing as ‘safe smoking’ and every method of tobacco consumption and every cigarette increases the risk for diseases [31,34]. Indeed, both classical and novel methods of smoking emit similar toxic compounds while tobacco is smoked [35]. Nitrosamines and polycyclic aromatic hydrocarbons—a major tobacco smoke compound—are carcinogenic and associated with cardiovascular complications [36]. In addition to this, e-cigarette liquid contains nicotine, propylene glycol and glycerine—precursors for toxic effects. During the process of heating up the liquid toxic aldehydes and ketones are formed as degradation products. These carbonyl compounds are a prime cause responsible for negative health effects. For example, the formed formaldehyde or acrolein is, due to oxidation stress and inflammation, toxic to proteins and DNA [37]. Furthermore, volatile organic and inorganic compounds like metals or carbon monoxide (CO) can be detected in both traditional and electronic cigarette smoke [20,37,38]. Moreover, in e-cigarette vapour (aerosol/mist produced by it) with nicotine, nitrosamines were detected [32]. CO is a toxin well-known for its ability to bind to hemoglobin and inhibit oxygen delivery [21].

Smoking is an established risk factor for heart failure and is independent of other traditional risk factors [1]. This is due to the exposure to carbon monoxide which causes oxidative stress, inflammation and mitochondrial dysfunction. This leads to endothelial dysfunction and exacerbated atherosclerosis and further to a decline in kidney function. Endothelial dysfunction leads to vascular disorders, a decrease in nitrogen monoxide production and an increase in the amount of reactive oxygen species and pro-inflammatory agents [2]. This further leads to an increase in vascular permeability and leukocyte adhesion. This is particularly important because the endothelium plays a particular role in cardiovascular homeostasis due to regulation of blood fluidity, fibrinolysis, vessel wall tension, angiogenesis or platelet aggregation [2]. Therefore, the endothelium can sometimes be compared to the guardian of cardiovascular health and its dysfunction is associated with various diseases such as atherosclerosis, hypertension, obesity, and diabetes mellitus. Besides endothelium, smoking can rapidly increase systolic and diastolic blood pressure, total systemic vascular resistance, pulmonary artery pressure and pulmonary vascular resistance [39]. It also contributes to an increase in the left ventricular mass of the heart [40]. These processes contribute to heart failure altogether. Obesity, diabetes mellitus II, atrial fibrillation, and dyslipidemia are all associated with smoking [41,42]. Smoking also increases the risk of ischemic heart disease. Cessation in smoking significantly decreases the risk of heart failure [3,42]. The meta-analysis of prospective studies by Aune et al. [3] reported a pooled relative risk of HF of 1.75 (95% CI 1.54–1.99) for current smokers, 1.44 (1.34–1.55) for ever-smokers, and 1.16 (1.08–1.24) for former smokers compared with never-smokers, with a clear dose–response of RR 1.41 (1.01–1.96) per 10 cigarettes/day; the risk after cessation declines by approximately 21% per 10 years since quitting (RR 0.79, 95% CI 0.63–1.00) [3]. Subsequent prospective data from the ARIC cohort [6], with *n =* 9345 and 1215 incident HF cases over a median 13 years of follow-up, showed that current versus never smoking was associated with adjusted hazard ratios of 2.28 (95% CI 1.67–3.10) for HFpEF and 2.16 (95% CI 1.55–3.00) for HFrEF, with clear dose–response per 10 pack-years (HR 1.16 [95% CI 1.12–1.20] for HFrEF and 1.14 [95% CI 1.11–1.16] for HFpEF) and a significantly elevated HF risk persisting up to 20 to 30 years after smoking cessation.

### 4.2. Alcohol Addiction

Alcohol was recognized as a cardiotoxin over a century ago. Consumption of about 80 g of alcohol daily (according to some studies > 200), for 5 years is associated with the risk of alcohol-induced cardiomyopathy [43,44,45]. This disease is associated with impairment and enlargement of the left ventricle, changes in the thickness of the ventricle’s wall and an increase in ventricular mass. The pathological mechanism of these abnormalities consists of reactive oxygen species and oxidative stress, apoptosis of myocardium cells, degeneration of proteins and changes in the fatty acids’ metabolism [44]. Some studies suggest that minor (less than 10 units per week) alcohol consumption may decrease the risk of heart failure or ischemic heart disease [7,46]. Other studies note the decrease in risk of type 2 diabetes and metabolic syndrome [7,44]. A possible mechanism for this outcome may be an increase in high-density lipoprotein concentrations, anti-inflammatory activity, or anticoagulation effect [7,47]. Some authors noticed that minor alcohol consumption increased adiponectin concentration—a peptide secreted by adipose tissue cells that is associated with glucose and fatty acid metabolism and has anti-inflammatory properties [48,49,50]. The dose–response meta-analysis of eight prospective studies by Larsson et al. [46] demonstrated a non-linear, J-shaped relationship between alcohol intake and HF risk. Pooled RRs versus non-drinkers were 0.90 (95% CI 0.84–0.96) at 3 drinks/week, 0.83 (0.73–0.95) at 7 drinks/week, and 0.90 at 14 drinks/week, with the protective association attenuating and reversing at higher intakes. These observational estimates should be interpreted with caution because of healthy-user and sick-quitter bias and unmeasured factors such as socioeconomic status, so they should not be read as a recommendation to drink alcohol. Consumption above approximately 80 g/day for ≥5 years remains a well-established cause of alcoholic cardiomyopathy [46].

### 4.3. Drugs (Cocaine, Amphetamine, Methamphetamine, Cannabis)

Cocaine has a high addictive potential and is one of the most commonly used drugs worldwide with an estimated number of users around 18 million [51,52]. Cocaine increases dopamine and serotonin uptake—neurotransmitters associated with pleasure perception [53,54]. Intoxication has particular consequences for the cardiovascular system that can be either chronic or acute. Major complications are composed of heart infarction, aortic dissection, cardiomyopathy, stroke, hypertension, chest pain, arrhythmia and heart failure. That is why endomyocardial biopsies performed on addicted people often reveal localized necrosis of cardiomyocytes and interstitial fibrosis [51,52,53,54,55]. Cocaine has negative inotropic activity due to sodium channel inhibition. This leads to the inhibition of sodium currents and a reduction in myocardial action potential (phase 0 depolarization) and intracardiac conduction. This prolongs the QRS interval and contributes to arrhythmia and sudden cardiac death. Moreover, disruption of myocardial conduction and contractility leads to impairment of left ventricular function and further to heart failure. Cocaine can also increase the amount of intracellular calcium, particularly in smooth muscle cells composing vesicular walls. It can influence the catecholamines’ metabolism by inhibiting presynaptic reuptake of these amines and increasing the secretion of amines in the nervous system. This further leads to overstimulation of alpha-1 receptors and contraction of smooth muscle cells. In addition, stimulation of heart beta-1 receptors leads to an increased risk of tachyarrhythmia and sudden cardiac death. Additionally, factors contributing to cocaine cardiotoxic activity consist of a decrease in coronary artery blood flow, an increase in thromboxane production, thrombocyte aggregation and increased activity of plasma activator 1 (PAI-1) in plasma. Cocaine-associated cardiomyopathy is also related to inhibition of potassium channels and disruption of calcium homeostasis. Equally important are ventricular hypertrophy and genetic factors. Regarding the epidemiology of cardiovascular complications in addicted populations, the exact prevalence of heart failure remains difficult to establish and varies significantly across studies. Depending on the study population and the screening methods used (e.g., self-reports vs. toxicology), reported rates of cocaine-related cardiac involvement range from 2.5% to over 20%. While some data suggest that approximately 5% of asymptomatic individuals may exhibit left ventricular systolic or diastolic dysfunction, these figures fluctuate depending on the diagnostic criteria applied [54,55,56,57]. Consumption of amphetamine and methamphetamine can also induce cardiomyopathy. The pathogenesis is multifactorial and composed of catecholamine secretion stimulation, constriction of coronary vessels, ischemia and direct toxicity [58,59]. This further leads to heart failure. The histological examination reveals necrosis, interstitial fibrosis, eosinophilic degeneration and vacuolization [60]. Similar to cocaine intoxication, the disruption of calcium homeostasis and the production of reactive oxygen species also play a role in disease induction [58]. The next discussed compound, cannabis, interacts with two types of receptors, and the effect of its use depends on the mode of ingestion and the amount of cannabinoid. The CB1 receptor is associated with negative influences on the cardiovascular system, endothelial dysfunction, oxidative stress and fibrosis. On the other hand, the CB2 receptor works quite contrarily. Consumed delta-9-tetrahydrocannabinol (Δ^9^-THC) activates both receptors and increases the risk of acute coronary syndrome and heart failure (irrespective of the occurrence of myocardial infarction) [61,62]. The meta-analysis of available cocaine-and-cardiomyopathy studies by Arenas et al., 2020 [24], found that chronic cocaine use is associated with significantly lower left ventricular ejection fraction and higher prevalence of left ventricular dysfunction compared with non-users. Reported HF prevalence among cocaine users ranges across analyzed studies from 2.5% to over 20%, against a baseline prevalence below 0.5% in matched age groups. In a National Readmissions Database analysis of 978,217 HF hospital admissions described by Thyagaturu et al. [25], patients with concomitant cocaine, amphetamine or cannabis use disorder (3.5% of the cohort) had a significantly higher risk of 30-day all-cause hospital readmission (adjusted hazard ratio 1.16, 95% CI 1.12–1.21). Moreover, in cannabis users a recent meta-analysis of 24 studies by Storck et al. [26] reported a pooled RR of cardiovascular mortality of 2.10 (95% CI 1.29–3.42) and an RR of 1.29 (95% CI 1.05–1.59) for acute coronary syndrome.

### 4.4. Caffeine

Tea and coffee are widely used worldwide and are the major sources of caffeine consumption and both play a role in cardiovascular prophylaxis. It is thought that moderate use of coffee and tea (2–3 cups per day) is associated with a positive influence on metabolic syndrome, hypertension and diabetes [63,64,65,66,67]. Though some studies suggest that it may increase the risk of dyslipidemia [63]. Coffee consumption is associated with a decrease in risk of diseases like heart failure, and arrhythmia, ischemic heart disease and decreases total cardiovascular risk [63,67]. Similarly, consumption of tea, particularly green tea, is associated with cardiovascular benefits and prolonged life expectancy if consumption is about 3 cups per day [68]. A cup of coffee consists of 95 mg of caffeine, whereas a cup of black and green tea has about 55 and 35 mg, respectively, and its half-life time is estimated to be 6 h, and it has 100% bioavailability [67,69]. Caffeine stimulates the secretion of adrenaline and noradrenaline and activation of the sympathetic nervous system. It is also associated with the inhibition of phosphodiesterase and an increase in cytosol calcium. Unfortunately, an increase in the intracellular calcium concentration may have a proarrhythmic effect [67]. Animal studies showed that high caffeine concentration (15 mg/kg) may induce ventricular fibrillation. On the other hand, other studies suggest that minor caffeine consumption may be related to an antiarrhythmic effect due to the inhibition of adenosine receptors [63,67]. Other positive aspects consist of an increase in endothelial NO secretion, vascular dilation and antioxidative effect [63,67]. It is possible that the use of coffee and tea is related to a decrease in heart failure risk and this was also observed in the Framingham Heart Study (FHS). The lowest occurrence was observed among participants consuming 2 cups per day. It is also possible that the use of caffeine will improve the condition of patients diagnosed with heart failure [1,63]. In addition, caffeine administered intravenously in a dose of 4 mg/kg (so like 2 cups) could increase the duration of average exercise and increase peak minute ventilation without affecting oxygen consumption [70]. Minor to moderate daily coffee consumption (1–4 cups per day) was associated with improved global longitudinal strain, which is a valuable tool used for screening for slight left ventricular dysfunction, and diastolic function on echocardiography compared to non-coffee drinkers. However, consumption of more than 4 cups was associated with a decrease in left ventricular ejection fraction [63]. Besides caffeine, tea and coffee also contain antioxidants contributing to cardioprotective effects [63,67,70]. A pooled machine-learning analysis of the Framingham Heart Study, ARIC and Cardiovascular Health Study cohorts by Stevens et al. [22] reported significant associations between habitual coffee intake and lower HF risk in CHS (HR 0.88 per cup/day, 95% CI 0.79–0.97). A similar trend was shown in ARIC (HR 0.98 per cup/day, 95% CI 0.96–1.00; *p* = 0.06). The HF risk was not significantly different between non-drinkers and participants consuming 1 cup/day, although it was reduced for those consuming 2 cups/day (HR 0.69, 95% CI 0.55–0.87) and ≥3 cups/day (HR 0.71, 95% CI 0.58–0.89) compared with non-drinkers. The dose–response meta-analysis by Mostofsky et al. [23] showed a J-shaped relationship with the lowest HF risk at approximately four servings/day and a possible reversal at very high intake (≥9–10 servings/day).

### 4.5. Sleep Disorders

Sleep disorders like insomnia or obstructive sleep apnea are associated with heart failure [71]. About 33% of patients with heart failure suffer from some kind of sleep disorder [72]. It was studied that these problems can increase the risk of patients’ death [71]. Problems such as short sleep duration, poor sleep quality and respiratory distress are widely associated with heart failure [71,72,73]. It should be emphasized that due to sleep disorders the overactivity of the sympathetic nervous system occurs [71]. Insomnia is the most common sleep disorder, occurring among 15% of the population and often accompanied by hypertension, coronary heart disease and heart failure though the exact etiopathology still remains uncertain. Insomnia can be associated with unhealthy behavioural habits and lack of physical activity and nutrition. It was studied that an unhealthy diet and a sedentary lifestyle contribute to an increase in insomnia symptoms [73]. This leads to a vicious circle, as sleep deprivation results in impaired daytime activity and fatigue which further contributes to a sedentary lifestyle. Obstructive sleep apnea (OSA) is caused by the recurring collapse of the upper respiratory tract during sleep, which leads to a further decrease in blood oxygen concentration [71]. Apnea and hypoventilation repeat many times each night. This contributes to increasing the risk of stroke, diabetes, hypertension, coronary heart disease and heart failure [71,72,73]. Moreover, there is a strong, two-way connection between sleep disorders and heart failure, because while sleep disturbances can trigger HF, undiagnosed HF can also present like a sleep disturbance [71,72]. Approximately one third of patients with established HF have clinically significant sleep-disordered breathing [72]. First symptoms of heart failure, such as night coughing and orthopnea are usually seen one year prior to HF diagnosis [71]. Successful HF interventions, for example heart transplantation, lead to the cure of co-existing conditions like sleep apnea [71]. The Sleep Heart Health Study by Gottlieb et al. [14] reported an adjusted HR for incident HF of 1.68 (95% CI, 1.02–2.76) in men with severe OSA (apnea–hypopnea index, AHI ≥ 30) compared with men with AHI < 5. More recent prospective meta-analysis (*n* > 25,000, median 9-year follow-up) by Craciun et al. [15] showed HR of 1.21 (95% CI, 0.98–1.50) for mild, 1.56 (95% CI, 1.20–2.03) for moderate and 2.45 (95% CI, 1.85–3.25) for severe OSA, with adherent CPAP therapy (≥4 h/night) reducing the cardiovascular risk to HR 0.76 (95% CI, 0.60–0.96). It is to be remembered that CPAP RCTs are heterogeneous on hard cardiovascular endpoints.

### 4.6. Stress

Psychological stress contributes to cardiovascular risk and can be considered partly modifiable. Stress-induced cardiomyopathy, also known as Takotsubo syndrome, can be caused by emotional and physical stress [74,75]. It leads to dilatation of the left ventricle and further heart failure. Symptoms of this disease are similar to those of acute coronary syndrome. Stress induces an adaptive physiological response characterized by increased hypothalamic–pituitary–adrenal axis activity, release of catecholamines and cortisol, and decreased activity of the vagus nerve. This leads to heart acceleration, an increase in cardiac output and blood pressure. Moreover, it leads to an increase in the secretion of pro-inflammatory cytokines and intensifies the thrombotic processes [75]. Chronic stress continuously stimulates those mechanisms. One of the important stressors can be a life-threatening disease like heart failure. It is currently studied that chronic exposure to stress can exacerbate heart failure [76]. The INTERHEART case–control study (*n =* 27,098 from 52 countries) [16] reported that the composite psychosocial index combining depression, perceived stress at work and home, financial stress and major life events was associated with an adjusted odds ratio of 2.67 (99% CI 2.21–3.22) for first myocardial infarction. The PURE prospective cohort [17] confirmed as well an independent association between high perceived stress and incident cardiovascular events.

### 4.7. Obesity

Obesity is defined as excessive accumulation or maldistribution of adipose tissue. Table 3 shows classification and comparative characteristics of anthropometric and instrumental obesity metrics.

Visceral obesity is defined as a waist circumference > 102 cm in men and >88 cm in women. In addition, obesity can be defined as visceral for a waist-to-hip ratio > 0.9 for men and >0.85 for women or gluteal-femoral obesity otherwise [77]. Obesity is a major heart failure risk factor particularly with preserved ejection fraction and contributes to hypertension and coronary heart disease. Obesity is also associated with diabetes mellitus 2, steatosis of the liver, stroke, dyslipidemia, hypertension and obstructive sleep apnea which are risk factors for heart failure [77]. Total blood volume and cardiac minute volume are correlated with being overweight. An increase in cardiac minute volume can be attributed to an increase in left ventricular stroke volume and work due to the fact that heart rate will remain the same, that is, for an ideal body mass. Severe obesity due to hemodynamic changes in the cardiovascular system contributes to the remodelling of the heart and leads to heart failure. The hypertrophy of the left ventricle in severe obesity can be eccentric (more common in normotensive individuals) or concentric (when obesity is accompanied by hypertension) [78,79]. Weight loss in severely obese patients is usually able to reverse most abnormalities related to it. However, patients with heart failure and minor overweight have extended life expectancy compared to patients with normal body weight—this is called an obesity paradox and the reasons for this phenomenon remain uncertain [80]. So far it is known that cachexia is associated with an unfavourable prognosis, and it is known that heart failure is associated with catabolism. Therefore, one theory suggests that patients with minor obesity might have greater metabolic reserves. Another theory suggests that some cytokines related to obesity may have a protective effect. Adipose tissue is known for its ability to secrete soluble receptors for TNF-α that may have a protective effect on chronic and acute heart failure related to neutralization of the factor itself. Moreover, obese patients may tend to have a weakened renin–angiotensin–aldosterone system [78,79,80]. Early robust evidence was provided by Hägg et al. [8], whose Mendelian-randomization (MR) analysis of *n =* 22,193 patients from 9 prospective cohorts showed a causal effect of adiposity on the development of heart failure (HR = 1.93 per SD increase in BMI, 95% CI, 1.12–3.30, *p* = 0.017). Moreover, the Copenhagen General Population Study and Copenhagen City Heart Study [9] reported an observational HR for incident HF of 1.06 (95% CI 1.05–1.07) per 1 kg/m^2^ increment in BMI, and Mendelian-randomization analyses showed a causal genetic RR of 1.39 (1.27–1.52) per 1 kg/m^2^, providing strong evidence that the relationship is rather causal than merely correlative.

### 4.8. Diet

Various diets have been developed to protect against cardiovascular diseases; their direct impact on prevention remains uncertain. An unbalanced, poor diet is a well-established factor in heart failure onset and progression probably due to impaired fatty acid metabolism and the increase in oxidative stress, which both can lead to atherosclerosis [81,82]. The Mediterranean diet and DASH diet (dietary approaches to stop hypertension) showed a positive influence on heart function and were associated with a lower risk of heart failure [81,82]. The term Mediterranean diet is used to describe traditional dietary habits of the 1960s of countries bordering the Mediterranean Sea like Greece, Italy, France and Spain. In these regions nutrition-related diseases were considered low with long life expectancy [81]. The diet is composed of regular consumption of plant-based foods (cereals, fruits, vegetables, legumes, nuts or seeds); moderate consumption of fish, seafood and dairy products; and low–moderate consumption of alcohol (mainly red wine) with relatively decreased consumption of red meat and other meat products [81]. Subsequently, the DASH diet is recommended for patients with hypertension. It is rich in antioxidants, micronutrients, fibre and whole grains, fruit and vegetables. It also contains low-fat dairy products, lean meat, poultry, fish, nuts, seeds and legumes. It also recommends decreasing the consumption of fats and oils [78]. Both diets can contribute to cardiovascular disease prevention; the scientific data should be analyzed with caution [81,82]. Recent meta-analysis of observational studies by Arayici et al. [12] reported that high adherence to the Mediterranean diet was associated with an odds ratio of 0.77 (95% CI 0.63–0.93) for incident HF and high adherence to the DASH diet with an odds ratio of 0.83 (0.70–0.98), compared with low adherence. Moreover, high Mediterranean diet adherence was also associated with lower all-cause mortality (OR 0.88, 0.78–0.99) among patients with established HF. A larger dose–response meta-analysis of eleven prospective cohorts *(n =* 450,451) by Yan et al. [13] showed an approximately 25% lower HF risk in the highest versus the lowest adherence to DASH, Mediterranean and Alternative Healthy Eating Index dietary patterns combined (HR of 0.75, 95% CI 0.67–0.84; *p* < 0.001). Dose–response analyses revealed linear associations between adherence to each dietary pattern and HF risk.

### 4.9. Physical Activity

It is well known that exercise is the best medicine, and movement can replace many drugs, but no drug can replace movement. Activity can be a heart failure prophylaxis and physical training is a part of treatment for patients with heart failure [83]. Exercise capacity is an important prognostic parameter for heart failure. It was proven in epidemiological studies that cardiorespiratory fitness is negatively correlated with cardiovascular risk [83,84,85]. Among cyclists of the Tour de France a reduction in mortality of up to 41% in comparison to the general population was reported [84]. When it comes to pathophysiological mechanisms, it is known that physical activity improves endothelial function by increasing nitric oxide bioavailability and decreasing oxidative stress [83]. Exercising is also known as a systemic anti-inflammatory intervention. Routine physical activity lowers the level of pro-inflammatory cytokines like TNF-α and IL-6 while increasing the level of protective mediators such as IL-10 [83]. Long-term exercise helps in reverse cardiac remodelling due to lowering afterload and reducing neurohormonal stress [83,85]. A dose–response meta-analysis of 29 prospective studies with 1 895 300 participants and 73 391 HF events by Aune et al. [10] reported a pooled relative risk of HF of 0.77 (95% CI 0.70–0.85) for high versus low total physical activity, 0.74 (95% CI 0.68–0.81) for leisure-time activity, and 0.71 (95% CI 0.65–0.78) per 20 MET-hours/week of leisure-time activity, with non-linear flattening of the curve at approximately 15–20 MET-hours/week. The summary RR for high cardiorespiratory fitness was 0.31 (95% CI 0.19–0.49). A separate non-occupational physical activity meta-analysis of 196 articles with over 30 million participants by Garcia et al. [11] reported a 27% lower CVD risk (95% CI 21–31%) at 8.75 mMET-hours/week, equivalent to the recommended 150 min/week of moderate-to-vigorous activity, with the steepest gradient between zero and ~150 min/week. However, the associations were weaker for the incidence of specific CVD outcomes such as coronary heart disease, heart failure and stroke. The association observed for heart failure was a 16% lower risk (HR 0.84; 95% CI (0.75–0.93)) at 8.75 mMET-hours/week.

### 4.10. Vitamin Deficiencies

Heart failure is often associated with vitamin B1 (thiamine) deficiency [86]. This may be related to diuretic treatment that increases vitamin excretion in urine [28]. Moreover, beriberi (a disease caused by B1 deficiency) may have a variant with heart failure [86]. The mechanism of high-output heart failure in beri-beri is connected to excessive vasodilatation and decreased peripheral vascular resistance. It is known that vitamin B1 is needed for proper ATP synthesis; the impaired cardiac energy metabolism leads to changes in vascular resistance and cardiac muscle vasodilation, as cardiac muscles require large amounts of ATP [86]. Although severe avitaminosis is rare in developed countries, a more subtle deficiency occurs secondary to long-term diuretic therapy, prolonged parenteral nutrition, dialysis or alcoholism and may contribute to congestive heart failure [28]. Other nutrients, particularly vitamins C and E or beta-carotene, due to their antioxidative effects, can have a protective impact on the cardiovascular system [87]. Moreover, vitamins B6, B12 and folic acid can decrease homocysteine concentration. Homocysteine contributes to atherosclerosis [88]. In other studies, supplementation with carnitine, coenzyme Q10 or creatine resulted in improved exercise capacity among participants with heart failure [4]. Furthermore, some studies suggest that vitamin D deficiency is related to the increased risk of heart failure among elderly people and studies suggest that up to 65% of them may have a deficiency of this vitamin in northern countries [5].

The mechanism behind vitamin D-related HF involves systemic inflammation due to insufficient calcitriol levels leading to the overactivation of pro-inflammatory pathways and an immune imbalance that favours pro-inflammatory Th17 cells over protective Treg cells [5]. Another important pathway that should be mentioned is cardiac remodelling, which is supposed to be provoked by the matrix metalloproteinases (specifically MMP-2 and MMP-9). Their activity leads to excessive extracellular matrix degradation, progressive ventricular dilatation, and finally to left ventricular hypertrophy. Metalloproteinases are usually inhibited through the calcitriol activity; vitamin D deficiency may cause heart failure [5]. A large meta-analysis of 80 RCTs (*n* = 163,131) by Ruiz-García et al. [27] found that vitamin D supplementation was associated with a small reduction in all-cause mortality (OR 0.95, 95% CI 0.91–0.99) but did not significantly reduce the incidence of heart failure. These findings suggest that low 25(OH)D may be a marker of poor general health rather than a directly modifiable driver of HF risk. Routine supplementation cannot currently be recommended as a strategy for HF prevention. Further clinical observations are needed. There are no large RCTs regarding other vitamin deficiencies.

### 4.11. Social Determinants of Behavioural Risk

Behavioural risk factors should not be analyzed without considering the social context in which they occur. The capacity of an individual to modify smoking, diet, physical activity or alcohol consumption is shaped by social determinants of health (SDOH), and ignoring this context risks oversimplifying behavioural risk and shifting responsibility to the patient [19].

Lower socioeconomic status (SES) is associated with higher prevalence of smoking, obesity, sedentary behaviour and poor diet, as well as higher heart failure incidence and mortality across the life course. A meta-analysis by Potter et al. [18] of 11 studies and over 6.3 million patients showed that low SES was associated with a 62% higher risk of incident HF (HR 1.62, 95% CI 1.50–1.76), with the strongest effect for income (HR 1.87, 95% CI 1.33–2.62), followed by education (HR 1.66, 95% CI 1.30–2.11) and occupation (HR 1.54, 95% CI 1.22–1.95). Moreover, factors such as neighbourhood walkability, access to green spaces, local food and air pollution can modify physical activity levels and dietary patterns regardless of individuals’ choices [89]. On the other hand, healthcare access, including primary care, affordability of pharmacotherapy, smoking-cessation services and availability of behavioural therapy determines whether identified risk is actually modified. This has been proven in the ARIC [19], where low income was associated with higher mortality HR 1.52 (95% CI, 1.14–2.04) and hospital readmission HR 1.45 (95% CI, 1.04–2.03).

Therefore, primary prevention of HF should rely not only on individual patient counselling, but also on environmental and policy-level interventions, such as tobacco taxation, urban planning that supports physical activity, and better access to preventive care.

## 5. Discussion

Despite the recent progress in understanding the behavioural, modifiable factors of heart failure, there are several unmet needs in clinical practice and research. The long-term cardiovascular impact of ENDS and other unconventional smoking methods seems to require further observation, and more studies are needed to clarify and compare the risk to traditional smoking in the development of HF. The common societal misunderstanding needs public highlighting, since nowadays the novel smoking methods are becoming more popular and therefore widely advertised and recommended. Another important factor, which is usually connected to smoking is excessive alcohol consumption and addiction. It should be emphasized, however, that although some studies suggest minor potential benefits of alcohol consumption, in light of current evidence alcohol consumption cannot be regarded as a preventive measure for cardiovascular diseases. Particularly, when considering other factors related to alcohol consumption such as tobacco use or unhealthy eating habits it will be clear why it is currently stated that consumption of alcohol without moderation and common sense should be avoided. Relatively similar could be considered the use of drugs, especially cannabis, which is usually seen as a mild, non-addictive drug with potentially positive health benefits. Although some studies suggest that cannabidiol (CBD) might have a cardioprotective effect [64], it should be highlighted that cannabis influence on the cardiovascular system is understandably harmful, due to evidence-based pathophysiological mechanisms of action. We recommend that further directions of research should focus on explaining doubts and unknowns about the potential cardioprotective effect of cannabis, with a priority on finding strong evidence and recommendations for the use of this substance. The use of other drugs listed above should also be taken under careful observation, which could help in earlier diagnosis and establishing treatment protocols in drug users, including addiction therapy as a matter of urgency.

Regarding coffee and tea consumption it should be highlighted that complete cessation of these beverages to avoid heart failure onset and progression is usually not needed. The current analyzed evidence enables the recommendation of moderate daily coffee and tea consumption without any increase in cardiovascular risk; a future large cohort study could be useful in determining safe doses.

Through the analysis of sleep disorder data, we noticed an important link between sleep disorders and heart failure. It seems important to consider insomnia or sleep apnea not only as an HF progression factor but also as a risk factor for upcoming heart failure onset. The data show that HF can be both the cause and the consequence of sleep pathology [71]. It seems that prioritizing the identification of sleep disorders in high-risk groups of patients is essential for improving heart failure management. We suggest that the routine use of digital sleep monitoring devices, such as personal wrist-bands or watches, in combination with routine check-ups could help achieve better clinical outcomes, such as earlier diagnosis. It also seems important to inform patients how sleep quality directly impacts their heart health.

Psychological stress remains an under-researched factor in heart failure onset and progression [14,15]. As proven in analyzed papers, stress leads to cardiac remodelling. Moreover, the management and treatment of HF could also be an important and exhausting factor for the patient. Many of the actual stressors are beyond the patient’s control, such as financial problems with covering treatment costs, caregiving duties, and cardiac rehabilitation. We suggest that healthcare systems must move toward care models that identify these problems and intervene directly; therefore the use of various forms of remote communication, such as video follow-ups or phone consults in high-risk groups could be helpful. Another useful intervention could be covering psychologists’ consultations, since better HF management can result in both longevity and an increase in quality of life.

Furthermore, the obesity paradox requires deeper investigation to explain the exact metabolic mechanisms that provide a survival advantage to some patients. This could lead to more personalized BMI targets for HF patients.

Our research proved once more that a healthy, balanced diet, rich in nutrients and antioxidants is a mandatory factor of cardiovascular health. Despite that established knowledge, many populations of patients suffer from diet-related diseases, whose onset and progression could be modifiable by changing eating habits. It should not only catch researchers’ and clinicians’ attention but also be turned into educational protocols. While educating and explaining diet modifications could collide with administering medication and analyzing test results in a simple follow-up visit in a doctor’s office, it seems to be achievable for a dietician or health educator specialist. We recommend creating health care teams for patients from high-risk groups, although it may cause problems with funding such protocols. Therefore it should be considered more as a wider challenge, including national health care providers. Further research could focus on calculating costs of care and comparing the results to costs of prophylaxis and health behaviour-promoting events.

Vitamin deficiencies are independent, novel risk factors, which are also diet-related. As mentioned above in this paper vitamin B1 deficiency requires further studies, which should focus on the relation between B1 supplementation and reduction in the occurrence of cardiovascular incidents including heart failure. Moreover, since other nutrients, particularly vitamins C, E, beta-carotene, folic acids, and vitamins B6 and B12 can have a protective impact on the cardiovascular system [4,18,89], it should be considered if additional supplementation of these compounds could be useful in high-risk patients.

Behavioural risk factors should not be analyzed without considering their social context. Important factors such as socioeconomic position, education level, built environment, access to primary care, smoking-cessation services and rehabilitation all together shape the prevalence of behavioural exposures [18,19,89]. It is important to take into consideration the patient’s abilities to modify such determinants. As described in Section 4.11 lower SES populations have higher HF incidence and mortality, and this gradient is observed even after adjustment for measured behavioural factors [19,89]. Therefore, effective primary prevention of HF cannot rely only on individual counselling but should also include more global interventions, such as tobacco and sugar-sweetened beverage taxation, smokefree legislation, public transport policies, and insurance coverage of preventive care. Moreover, healthcare systems should perform routine screening for social needs, including food insecurity and housing and transportation barriers, with cardiovascular risk assessment.

Regular physical activity induces functional, structural, cellular and molecular adaptations in the heart and even a small amount of exercise is better than none [8,79,80]. Since regular physical activity is crucial not only for prolonging HF onset but also for faster recovery, promoting exercises and mild physical activities such as walks, Nordic walking etc. could be useful to affected patients and serve their longevity. Moreover, administering exercise training with dedicated medication in patients from high-risk HF groups could augment existing treatment protocols and result in lowering medication dosages with better therapeutic outcomes. Healthcare professionals should focus on promoting physical activity, taking into consideration their own physical fitness, since it helps build trust in the patient–specialist relationship.

The behavioural risk factors discussed in this review can also be linked to established risk-prediction tools and primary-prevention frameworks [31]. The American Heart Association PREVENT^TM^ equations [31,90] include heart failure as both a 10- and a 30-year outcome and also use the estimated glomerular filtration rate, and optionally the urine albumin–creatinine ratio, HbA1c and a Social Deprivation Index when available. Within the PREVENT^TM^ framework smoking is included directly, while obesity, dietary patterns, alcohol consumption and physical activity act through body-mass index, blood pressure, lipid profile, eGFR and HbA1c levels. Psychological stress and the social determinants of behavioural risk are partially counted by the Social Deprivation Index. Several behavioural and emerging exposures discussed in this review, such as sleep disorders, ENDS, cannabis, illicit psychostimulants, high-dose caffeine, and vitamin B1 and D deficiencies, are not yet represented in PREVENT^TM^. These factors probably will not be incorporated until more robust prospective data are available. Future iterations of risk equations or complementary tools may need to capture these determinants, especially for younger adults, in whom the prevalence of such exposures is rising. In Table 4 we highlighted the clinical targets and intervention strategies for modifiable behavioural risk factors in heart failure alongside the expected impact on HF prevention. 

## 6. Conclusions

This review highlights that while the epidemiology of heart failure is established and known, the role of modifiable risk factors is evolving. Proper recognition of these factors, as presented in this review is necessary for developing effective treatment and prevention strategies. Primary prevention of HF should focus more on a holistic strategy, including not only physicians but also specialists of other health care domains. The evidence supporting individual risk factors is not uniform: tobacco smoking, obesity and physical inactivity are supported by Level A evidence, including Mendelian-randomization data. In contrast, evidence for the emerging exposures, such as ENDS, cannabis, high-dose caffeine and illicit psychostimulants, remains mainly observational and requires further high-quality prospective studies. It should also be highlighted that behavioural risk does not occur outside a structural context, since socioeconomic status, education, the environment and access to preventive care strongly influence the modifiability of these factors. Effective prevention will require structural and policy-level interventions but also individual-level counselling. Established risk-prediction tools such as the AHA PREVENT^TM^ could be useful in clinical decision-making. Future research should focus on an urgent need for clinical trials targeting reversible behavioural factors with hard HF endpoints to develop better clinical guidelines.

## Figures and Tables

**Table 1:** Quantitative effect estimates for behavioural HF risk factors. Reported as RR, HR, or OR according to study design and evidence grade (defined in Section Assessment of Evidence Quality). Bracketed citations refer to the manuscript.

Risk Factor	Quantitative Effect Estimate	Evidence Level/Study Design	Source
**Established risk factors**			
**Tobacco smoking**	Current smokers vs. never: RR 1.75 (95% CI 1.54–1.99) [3] Former smokers: RR 1.16 (95% CI 1.08–1.24) [3] Dose–response: RR 1.41 (95% CI 1.01–1.96) per 10 cigarettes/day [3] Risk decline after cessation: RR 0.79 (95% CI 0.63–1.00) per 10 years since quitting [3] current vs. never smoking: HR 2.28 (95% CI 1.67–3.10) for HFpEF and 2.16 (95% CI 1.55–3.00) for HFrEF [6]	**Level A** *Meta-analysis of prospective studies; cohort study (ARIC)*	Aune et al., Eur J Prev Cardiol 2019 [3] Ding et al., J Am Coll Cardiol 2022 [6]
**Excessive alcohol consumption**	Dose–response (J-shape) vs. non-drinkers: - 3 drinks/week: RR 0.90 (95% CI 0.84–0.96) - 7 drinks/week: RR 0.83 (95% CI 0.73–0.95) - 14 drinks/week: RR 0.90 (95% CI 0.73–1.10) - 21 drinks/week: 1.07 (95% CI 0.77–1.48) Caveat: healthy-user/sick-quitter bias	**Level B** *Dose–response meta-analysis of prospective cohorts*	Larsson et al., Eur J Heart Fail 2015 [7]
**Obesity**	Causal effect of adiposity on development of heart failure (HR = 1.93 per SD-increase in BMI, 95% CI, 1.12–3.30, *p* = 0.017) [8] Observational HR for HF incidence per 1 kg/m^2^ BMI: 1.06 (95% CI 1.05–1.07) [9] Mendelian randomisation causal RR for HF incidence per 1 kg/m^2^: 1.39 (95% CI 1.27–1.52) [9]	**Level A** *Observational + Mendelian randomisation, meta-analysis*	Hägg et al. Int. J. Epidemiol. [8] Benn et al., Cardiovasc Res 2023 [9]
**Physical inactivity**	High vs. low activity reduces HF risk by 23% (RR 0.77; 95% CI 0.70–0.85); non-linear benefit plateaus at 15–20 MET-h/week [10] High fitness level shows a massive 69% risk reduction for HF (RR 0.31; 95% CI 0.19–0.49) [10] Dose–response: Meta-analysis of >30 M people 150 min/week of moderate activity (8.75 mMET-h/week) reduces HF incidence by 16% [11]	**Level A** *Dose–response meta-analysis of 29 prospective studies*	Aune et al., Eur J Epidemiol 2021 [10] Garcia et al., Br J Sports Med 2023 [11]
**Unbalanced diet (low adherence to Mediterranean/DASH)**	Mediterranean diet associated with significant reduction in incident HF (OR 0.77; 95% CI 0.63–0.93) and lower all-cause mortality in established HF (OR 0.88; 0.78–0.99) [12] DASH diet proved high adherence associated with reduced HF risk (OR 0.83; 95% CI 0.70–0.98) [12] Dose–response meta-analysis (*n* = 450,451) showed 25% lower HF risk (HR 0.75; 95% CI 0.67–0.84) for highest vs. lowest adherence to combined healthy eating patterns [13]	**Level A–B** *Meta-analyses of prospective cohorts; limited RCT data on hard clinical endpoints for primary prevention*	Arayici et al., Life 2025 [12] Yan et al., Eur J Clin Nutr 2026 [13]
**Sleep disorders (obstructive sleep apnea, insomnia)**	Severe OSA (AHI ≥ 30 vs. AHI < 5), in men: HR 1.68 (95% CI, 1.02–2.76) [14] Pooled HR for incident HF (4 cohorts): 1.78 (95% CI 1.24–2.55) [14] Severity gradient (pooled HR for CV events) [15]: - Mild: 1.21 (95% CI 0.98–1.50) - Moderate: 1.56 (95% CI 1.20–2.03) - Severe: 2.45 (95% CI 1.85–3.25) CPAP ≥ 4 h/night: HR 0.76 (95% CI 0.60–0.96) [15]	**Level B–C** *Prospective cohorts, meta-analysis of prospective cohorts, review*	Gottlieb et al., Circulation 2010 [14] Craciun et al., Medicina 2025 [15]
**Psychological stress**	Composite Psychosocial Index (depression, stress, life events): Adjusted OR 2.67 (99% CI 2.21–3.22) for first MI [16] High stress was associated with CVD (HR, 1.22 (95% CI, 1.08–1.37)) [17]	**Level B–C** *Large case–control (INTERHEART),* *prospective cohort* *(PURE),* *narrative review*	Yusuf et al., Lancet 2004 (INTERHEART) [16] Santosa et al., JAMA Netw Open 2021 (PURE) [17]
**Adverse social determinants of health**	Low SES associated with a 62% higher risk of incident HF HR 1.62, 95% CI 1.50–1.76), for income HR 1.87, 95% CI 1.33–2.62), for education HR 1.66, 95% CI 1.30–2.11, for occupation HR 1.54, 95% CI 1.22–1.95 [18] low income was associated with higher mortality in HF patients HR 1.52 (95% CI, 1.14–2.04) and hospital readmission HR 1.45 (95% CI, 1.04–2.03) [19]	**Level B** *Systematic review and meta-analysis of 11 prospective cohorts, single-cohort data from ARIC*	Potter et al., Eur Heart J Qual Care Clin Outcomes 2019 [18] Mathews et al., J Am Heart Assoc 2022 [19]
**Emerging determinants**			
**Electronic nicotine delivery systems (ENDS)**	No reliable HF-specific RR/HR available Short-term endothelial dysfunction documented in mechanistic and small clinical studies	**Level C** *Mechanistic and small observational studies; long-term cohorts pending*	Auschwitz et al., Cells 2023 [20] Dorey et al., Mil Med 2020 [21]
**Caffeine (coffee/tea)**	Reduced risk of HF per cup/day: HR 0.95 (95% CI, 0.91–0.99) [22] 2 cups/day vs. none: HR 0.69 (95% CI, 0.55–0.87) [22] ≥3 cups/day vs. none: HR 0.71 (95% CI, 0.58–0.89) [22] J-shape: nadir ≈ 4 servings/day; possible reversal at ≥9–10 servings/day [23]	**Level B** *Pooled prospective cohorts (FHS, ARIC, CHS) with machine-learning analysis; dose–response meta-analysis*	Stevens et al., Circ Heart Fail 2021 [22] Mostofsky et al., Circ Heart Fail 2012 [23]
**Drugs (cocaine, amphetamine, methamphetamine, cannabis)**	HF prevalence among cocaine users: 2.5–20% (vs. <0.5% baseline in matched age groups) Chronic cocaine use: significantly lower LVEF and higher prevalence of LV dysfunction vs. non-users [24] Cocaine/amphetamine/cannabis use disorders + HF: aHR 1.16 (95% CI 1.12–1.21) for 30-day all-cause readmission (*n =* 978,217 HF hospitalisations; SUD prevalence 3.5%) [25] cardiovascular mortality among cannabis users vs. non-users: RR 2.10 (95% CI 1.29–3.42) [26] acute coronary syndrome among cannabis users: RR 1.29 (95% CI 1.05–1.59) [26]	**Level B–C** *Narrative review, systematic review/ meta-analyses,* *large retrospective cohorts*	Storck et al., Heart 2025 [26] Thyagaturu et al., Curr Probl Cardiol 2023 [25] Arenas et al., Sci Rep 2020 [24]
**Vitamin deficiencies (B1, D)**	Vitamin D deficiency in elderly: ↑ risk of incident HF (effect sizes vary) [5] Vitamin D supplementation was associated with a small reduction in all-cause mortality (OR 0.95, 95% CI 0.91–0.99) but did not significantly reduce the incidence of heart failure [27] Thiamine deficiency in HF on long-term loop diuretic therapy: prevalent and reversible with supplementation [28]	**Level A–C** *Consistent observational associations across cohorts and meta-analyses;* *large RCT meta-analyses do not support a causal role in HF prevention*	Porto et al., ESC Heart Fail 2018 [5] Sica, Congest Heart Fail 2007 [28] Ruiz-García et al., *Nutrients* 2023 [27]

Abbreviations: AHI, apnea–hypopnea index; ARIC, Atherosclerosis Risk in Communities; BMI, body mass index; CHS, Cardiovascular Health Study; CI, confidence interval; CPAP, continuous positive airway pressure; DASH, Dietary Approaches to Stop Hypertension; ENDS, electronic nicotine delivery systems; FHS, Framingham Heart Study; HF, heart failure; HFpEF/HFrEF, heart failure with preserved/reduced ejection fraction; HR, hazard ratio; LV, left ventricle; LVEF, left ventricular ejection fraction; MET, metabolic equivalent of task; MI, myocardial infarction; MR, Mendelian randomization; OR, odds ratio; OSA, obstructive sleep apnea; RCT, randomized controlled trial; RR, relative risk; SD, standard deviation.

**Table 2:** Modifiable heart failure risk factors and their clinical impact.

Category	Risk Factor	Main Pathophysiological Mechanisms	Clinical Impact and Evidence
**Established risk factors**	Tobacco smoking (traditional)	Oxidative stress, CO-induced mitochondrial dysfunction, endothelial inflammation	Increased left ventricular mass, exacerbated atherosclerosis, vascular disorders
	Excessive alcohol consumption	ROS, oxidative stress, apoptosis of myocytes, impaired fatty acid metabolism	Alcohol-induced cardiomyopathy, left ventricle enlargement and impairment
	Obesity	Cardiac remodelling, increased blood volume, increased cardiac minute volume	Eccentric or concentric hypertrophy of the left ventricle
	Physical inactivity	Increase in insulin resistance, increase in oxidative stress, chronic endothelial inflammation, mitochondrial dysfunction	Myocardial fibrosis, enlargement and impairment of the left ventricle
	Unbalanced diet	Increase in oxidative stress, impaired fatty acid metabolism	Higher risk of HF development and cardiac malfunction
	Sleep disorders (apnea, insomnia)	Recurring hypoxia, systemic inflammation, sympathetic nervous system overactivity	Increased mortality, faster HF progression
	Stress exposure	HPA axis overactivity, high catecholamine release, pro-inflammatory cytokine secretion	Takotsubo syndrome, LV dilatation, faster HF progression
**Emerging Determinants**	ENDS	Exposure to toxic compounds, oxidative stress, DNA damage, endothelial dysfunction	Potential cardiac involvement
	Cannabis	Endothelial dysfunction, oxidative stress, fibrosis	Increased risk of acute coronary syndrome and HF
	High-dose caffeine	Phosphodiesterase inhibition, sympathetic activation, increased intracellular calcium concentration	Potential proarrhythmic effect, decreased LVEF
	**Psychostimulants** (cocaine, amphetamine, methamphetamine)	Sodium/potassium channel inhibition, localized cardiomyocyte necrosis, increased thromboxane production and aggregation, increased PAI-1 activity	Decreased coronary arteries blood flow, arrhythmias, sudden cardiac death, systolic/diastolic dysfunction
	**Vitamin deficiencies** (B1, D)	ATP deficiency, endothelial dysfunction, oxidative stress	Cardiac enlargement, increased risk of acute coronary syndrome and HF

Abbreviations: ATP, adenosine triphosphate; CO, carbon monoxide; ENDS, electronic nicotine delivery systems; LV, left ventricle; PAI-1, plasma activator 1; LVEF, left ventricular ejection fraction.

**Table 3:** Classification and comparative characteristics of anthropometric and instrumental obesity metrics.

Obesity Metric	Classification	Definition	Cut-Off Points for Obesity	Characteristics
**Body Mass Index (BMI)**	Anthropometric	$\frac{Weight\;\;}{{Height}^{2}\;\left(m^{2}\right)}$ (kg/m^2^)	≥30.0 (kg/m^2^)	Standard screening tool, universally standardized, ignores fat distribution and the differences between lean muscle mass and fat mass
**Wst Circumference (WC)**	Anthropometric	Measurement around the waist (cm)	Men > 102 cm Women > 88 cm	Measures central adiposity, a better predictor of cardiovascular risk and metabolic syndrome than BMI, although it is sensitive to measurement errors
**Waist-to-Hip Ratio (WHR)**	Anthropometric	$\frac{Waist\;circumference\;\;}{Hip\;circumference\;\left(cm/in\right)\;}$ measurements must be in the same units	Men > 0.90 Women > 0.85	Assesses body fat distribution, excellent for identifying the apple-shaped body type associated with higher cardiovascular risk and mortality
**Waist-to-Height Ratio (WHtR)**	Anthropometric	$\frac{Waist\;circumference\;\;}{\;Height\;\left(cm/\;in\right)}$ measurements must be in the same units	>0.50 for both men and women	Equivalent to or slightly better than WC and superior to BMI in predicting higher cardiometabolic risk
**dual energy X-ray absorptiometry (DXA)**	Instrumental	Uses two distinct low-dose X-ray beams to quantify bone mineral content, lean tissue mass and fat mass, therefore calculates body fat percentage and visceral adipose tissue	No universal WHO consensus, common clinical cut-offs: Men > 25% body fat Women > 35% body fat	Extremely accurate and highly detailed exact body composition measurement
**Bioelectrical Impedance Analysis (BIA)**	Instrumental	Measures the resistance (impedance) of body tissues to an electrical current; lean, water-rich tissue conducts well, while fat mass resists. Used to estimate body fat percentage	No universal WHO consensus, common clinical cut-offs: Men > 25% body fat Women > 35% body fat	Inexpensive, rapid, non-invasive, widely accessible compared to DXA but less accurate; results are highly sensitive to hydration status, recent food intake, and exercise, can not accurately separate visceral from subcutaneous fat tissue

**Table 4:** Clinical targets and intervention strategies for modifiable behavioural risk factors in heart failure.

Behavioural Risk Factor	Target Goal	Primary Intervention Strategy	Expected Impact on HF Prevention
**Unhealthy diet**	Adherence to DASH or Mediterranean diet; Sodium restriction (<2–3 g/day)	Dietary counselling, nutritional education, legal restriction of ultra-processed foods	Reduces blood pressure, reduces systemic inflammation, helps to prevent obesity-related cardiac remodelling
**Sedentary lifestyle**	150 min/week of moderate-intensity or 75 min/week of vigorous aerobic exercise	Personalized exercise prescription, pedometer tracking, cardiac rehabilitation programmes	Improves endothelial function, enhances insulin sensitivity, prevention of left ventricular stiffness
**Smoking/tobacco use**	Complete cessation of smoking, avoidance of second-hand smoke	Behavioural therapy (CBT), nicotine replacement therapy, brief physician advice at every visit	Oxidative stress and endothelial damage reduction, lowering the risk of coronary artery disease
**Heavy alcohol consumption**	Abstinence or strict limitation	Screening (e.g., AUDIT tool, CAGE test), psychological support, addiction counselling	Prevents direct alcohol-induced myocardial toxicity, better blood pressure control
**Chronic stress and sleep deprivation**	>7 h of quality sleep per night; effective stress management	Cognitive Behavioural Therapy for Insomnia (CBT-I), mindfulness courses, sleep hygiene education, sleep diaries, CPAP for diagnosed OSA	Reduction in sympathetic nervous system (SNS) overactivation, resting heart rate decrease
**Obesity**	BMI 18.5–24.9 kg/m^2^; WC < 102 cm for men/<88 cm for women ≥5–10% body-weight loss in adults with obesity	Multicomponent lifestyle intervention (diet + physical activity + behavioural counselling); Pharmacotherapy: GIP, GLP-1 receptor agonists, naltrexone/bupropione, phentermine/topiramate depending on dominant obesity phenotype bariatric surgery in selected high-risk patients	Reversion of concentric/eccentric LV remodelling, lowering cardiac output overload, attenuating HFpEF risk
**Drugs**	Complete abstinence; harm reduction in those unable to abstain	Screening at primary-care visits, referral to addiction services, cognitive-behavioural therapy and contingency management, treatment of comorbid mental-health disorders	Prevention of direct cardiotoxicity, coronary spasm, stimulant-induced cardiomyopathy and 30-day HF hospital readmissions
**Caffeine**	Moderate consumption (≤3–4 cups of coffee/day), energy drinks and high-dose caffeine supplements avoidance	Patient education, review of dietary supplements at primary care visits	Cardioprotective benefit at moderate doses while avoiding proarrhythmic effects at very high intake (≥9–10 servings/day)
**ENDS**	Complete cessation do not use ENDS as a long-term substitute for smoking cessation	Behavioural counselling, evidence-based cessation pharmacotherapy, public-health regulation of flavoured products and youth marketing	Reduction in exposure to ultrafine particles, heavy metals, and carbonyl compounds with documented endothelial effects
**Vitamins**	Adequate dietary intake; correction of documented deficiency screening of HF patients on long-term loop diuretic therapy	Targeted thiamine supplementation in HF patients on chronic loop diuretics Adequate serum levels of vitamin D supplementation (no benefit shown for routine supplementation in HF prevention)	Restoration of ATP-dependent myocardial energy metabolism (thiamine)
**Adverse social determinants of health**	Equitable access to primary care, cardiac rehabilitation and smoking-cessation services safe and walkable built environments affordable healthy foods	Integration of SDOH screening into clinical workflows multilevel public-health policy e.g., taxation of tobacco/alcohol/sugar-sweetened beverages, health-insurance coverage) community-based interventions	Reduction in health inequalities in HF incidence and outcomes

Abbreviations: AUDIT, Alcohol Use Disorders Identification Test; BMI, body mass index; CAGE, Cut down/Annoyed/Guilty/Eye-opener questionnaire; CBT, cognitive behavioural therapy; CBT-I, cognitive behavioural therapy for insomnia; CPAP, continuous positive airway pressure; DASH, Dietary Approaches to Stop Hypertension; ENDS, electronic nicotine delivery systems; GIP, Gastric inhibitory polypeptide; GLP-1, glucagon-like peptide 1; HF, heart failure; HFpEF, heart failure with preserved ejection fraction; LV, left ventricular; OSA, obstructive sleep apnea; SDOH, social determinants of health.

## Data Availability

No new data were created or analyzed in this study.

## Abbreviations

The following abbreviations are used in this manuscript:

AHA	American Heart Association
AHEI	Alternative Healthy Eating Index
AHI	Apnea–hypopnea index
ANP	Atrial natriuretic peptide
ARIC	Atherosclerosis Risk in Communities (study)
ATP	Adenosine triphosphate
AUDIT	Alcohol Use Disorders Identification Test
BIA	Bioelectrical impedance analysis
BMI	Body Mass Index
BNP	Brain natriuretic peptide
CAGE	Cut down/Annoyed/Guilty/Eye-opener questionnaire
cAMP	Cyclic adenosine monophosphate
CB1	Cannabinoid receptor 1
CB2	Cannabinoid receptor 2
CBD	Cannabidiol
CBT	Cognitive behavioural therapy
CBT-I	Cognitive behavioural therapy for insomnia
CHS	Cardiovascular Health Study
CI	Confidence interval
CO	Carbon monoxide
CPAP	Continuous positive airway pressure
CV	Cardiovascular
CVD	Cardiovascular disease
DASH	Dietary Approaches to Stop Hypertension
DNA	Deoxyribonucleic acid
DXA	Dual-energy X-ray absorptiometry
EF	Ejection Fraction
ENDS	Electronic nicotine delivery systems
ESC	European Society of Cardiology
ET-1	Endothelin-1
FHS	Framingham Heart Study
GFR	Glomerular filtration rate
GLP-1	Glucagon-like peptide 1
HF	Heart Failure
HFimpEF	Heart Failure with improved Ejection Fraction
HFmrEF	Heart Failure with mildly reduced Ejection Fraction
HFpEF	Heart Failure with preserved Ejection Fraction
HFrEF	Heart Failure with reduced Ejection Fraction
HPA	Hypothalamic–pituitary–adrenal axis
HR	Hazard ratio
IL-6	Interleukin 6
IL-10	Interleukin 10
INTERHEART	INTERHEART Study
LoE	Level of evidence
LV	Left Ventricle
LVEF	Left Ventricular Ejection Fraction
MAP	Mean Arterial Pressure
MET	Metabolic equivalent of task
MI	Myocardial infarction
MMP-2	Matrix metalloproteinase-2
MMP-9	Matrix metalloproteinase-9
MR	Mendelian randomisation
NO	Nitric oxide/Nitrogen monoxide
NP	Natriuretic Peptide
OR	Odds ratio
OSA	Obstructive sleep apnea
PAI-1	Plasminogen activator inhibitor-1
PAR	Population-attributable risk
PREVENT	Predicting Risk of cardiovascular disease EVENTs (AHA equation)
PURE	Prospective Urban Rural Epidemiology study
QRS	QRS complex (electrocardiographic)
RAAS	Renin–Angiotensin–Aldosterone System
RCT	Randomized controlled trial
ROS	Reactive oxygen species
RR	Relative risk
SD	Standard deviation
SDB	Sleep-disordered breathing
SDOH	Social determinants of health
SES	Socioeconomic status
SNS	Sympathetic Nervous System
SUD	Substance use disorder
Th17	T helper 17 cells
TNF-α	Tumour necrosis factor alpha
Treg	Regulatory T cells
WC	Waist circumference
WHO	World Health Organization
WHR	Waist-to-hip ratio
WHtR	Waist-to-height ratio
Δ^9^-THC	Delta-9-tetrahydrocannabinol
25(OH)D	25-hydroxyvitamin D
